# The ACUTE (Ambulance CPAP: Use, Treatment effect and economics) feasibility study: a pilot randomised controlled trial of prehospital CPAP for acute respiratory failure

**DOI:** 10.1186/s40814-018-0281-9

**Published:** 2018-06-18

**Authors:** Gordon W. Fuller, Steve Goodacre, Samuel Keating, Gavin Perkins, Matthew Ward, Andy Rosser, Imogen Gunson, Joshua Miller, Mike Bradburn, Praveen Thokala, Tim Harris, Andrew Carson, Maggie Marsh, Cindy Cooper

**Affiliations:** 1 Centre for Urgent and Emergency Care Research, School of Health and Related Research, University of Sheffield, Regent Court, 30 Regent Street, Sheffield, S1 4DA UK; 2 Clinical Trials and Research Unit, School of Health and Related Research, University of Sheffield, Regent Court, 30 Regent Street, Sheffield, S1 4DA UK; 3 Warwick Clinical Trials Unit, University of Warwick, Coventry, CV4 7AL UK; 4 West Midlands Ambulance Service, Trust Headquarters, Millennium Point, Waterfront Business Park, Waterfront Way, Brierley Hill, West Midlands, DY5 1LX UK; 5 Health Economics and Decision Science, School of Health and Related Research, University of Sheffield, Regent Court, 30 Regent Street, Sheffield, S1 4DA UK; 6 Blizard Institute, Barts and The London School of Medicine and Dentistry, 4 Newark Street, London, E1 2AT UK; 7 Sheffield Emergency Care Forum, Clinical Research Office Sheffield, Royal Hallamshire Hospital, D Floor, Glossop Road, Sheffield, S10 2JF UK

**Keywords:** Acute respiratory failure, Continuous positive airways pressure, Prehospital, Emergency medical services

## Abstract

**Background:**

Acute respiratory failure (ARF) is a common and life-threatening medical emergency. Standard prehospital management involves controlled oxygen therapy and disease-specific ancillary treatments. Continuous positive airway pressure (CPAP) is a potentially beneficial alternative treatment that could be delivered by emergency medical services. However, it is uncertain whether this treatment could work effectively in United Kingdom National Health Service (NHS) ambulance services and if it represents value for money.

**Methods:**

An individual patient randomised controlled external pilot trial will be conducted comparing prehospital CPAP to standard oxygen therapy for ARF. Adults presenting to ambulance service clinicians will be eligible if they have respiratory distress with peripheral oxygen saturation below British Thoracic Society (BTS) target levels, despite titrated supplemental oxygen. Enrolled patients will be allocated (1:1 simple randomisation) to prehospital CPAP (O_two system) or standard oxygen therapy using identical sealed boxes. Feasibility outcomes will include incidence of recruited eligible patients, number of erroneously recruited patients and proportion of cases adhering to allocation schedule and treatment, followed up at 30 days and with complete data collection. Effectiveness outcomes will comprise survival at 30 days (definitive trial primary end point), endotracheal intubation, admission to critical care, length of hospital stay, visual analogue scale (VAS) dyspnoea score, EQ-5D-5L and health care resource use at 30 days. The cost-effectiveness of CPAP, and of conducting a definitive trial, will be evaluated by updating an existing economic model. The trial aims to recruit 120 patients over 12 months from four regional ambulance hubs within the West Midlands Ambulance Service (WMAS). This sample size will allow estimation of feasibility outcomes with a precision of < 5%. Feasibility and effectiveness outcomes will be reported descriptively for the whole trial population, and each trial arm, together with their 95% confidence intervals.

**Discussion:**

This study will determine if it is feasible, acceptable and cost-effective to undertake a full-scale trial comparing CPAP and standard oxygen treatment, delivered by ambulance service clinicians for ARF. This will inform NHS practice and prevent inappropriate prehospital CPAP adoption on the basis of limited evidence and at a potentially substantial cost.

**Trial registration:**

ISRCTN12048261. Registered on 30 August 2017. http://www.isrctn.com/ISRCTN12048261

## Background

Acute respiratory failure (ARF) is a common medical emergency which occurs when disease of the heart or lungs lead to failure to maintain adequate blood oxygen levels and/or increased blood carbon dioxide levels [1]. It is caused by a number of common cardiac or respiratory diseases, including heart failure, pneumonia, and exacerbations of chronic obstructive pulmonary disease (COPD) and asthma [2]. There are approximately 3000 ARF cases in England per year, with a high 14% risk of death within 30 days [3]. ARF has substantial health services costs, with patients often requiring prolonged hospital stays, ventilatory support and critical care admissions [4]. ARF was responsible for over 3 million National Health Service (NHS) bed days in England in 2014 [5].

Current prehospital clinical practice guidelines recommend a standard management approach of oxygen therapy for the treatment of acute respiratory failure, supplemented by specific management options directed at the underlying disease [6–8]. Prehospital administration of continuous positive airway pressure (CPAP) may be a potentially beneficial alternative treatment strategy [9]. CPAP involves delivering oxygen-enriched air to the lungs at increased pressure through a close-fitting face mask and is widely used in hospital to treat ARF from a number of causes [10]. It has been suggested that CPAP may be more effective if delivered earlier, i.e. en route to hospital [11]. The difficulties of prehospital diagnosis mean that prehospital CPAP is likely to be applied generally to all cases of acute respiratory failure, rather than directed towards those due to a specific cause [12].

A recent evidence synthesis reported that prehospital CPAP reduced the risk of mortality and requirement for endotracheal intubation in ARF compared to standard treatment but noted that the primary studies were relatively small, heterogeneous, at risk of bias and may not be applicable to the NHS [3, 9]. A recent economic evaluation suggested that prehospital CPAP was more effective than standard care but was also more expensive, with an incremental cost-effectiveness ratio of £20,514/quality-adjusted life year (QALY) and a 49.5% probability of being cost-effective at the £20,000/QALY threshold. Expected value of perfect information (EVPI) analyses suggested that further research costing up to £22.5 million could represent value for money, while expected value of sample information (EVSI) analyses suggested that a randomised trial recruiting 1000 patients per arm would be cost-effective if research costs were less than £18.1 million. However, these cost-effectiveness results were predicated on the accuracy of published effectiveness data and were very sensitive to estimates for the incidence of acute respiratory failure.

Taken together, these findings suggest that although prehospital CPAP is a promising therapy, further research is needed to examine whether the reported clinical and cost-effectiveness are confirmed in the UK setting. Prior to a large pragmatic trial and economic evaluation comparing prehospital CPAP to standard care, it is first necessary to estimate the incidence of eligible patients and to assess whether a trial would be feasible and cost-effective. We also need to determine whether prehospital CPAP can be delivered in the context of the NHS ambulance service. Prehospital trials need to overcome a number of potential practical barriers if they are to deliver valid data. For these reasons, a stand-alone feasibility study is necessary to estimate the incidence of eligible patients and test the feasibility and acceptability of potential definitive trial methods.

## Methods

### Study design, aims and objectives

**Fig. 1:**
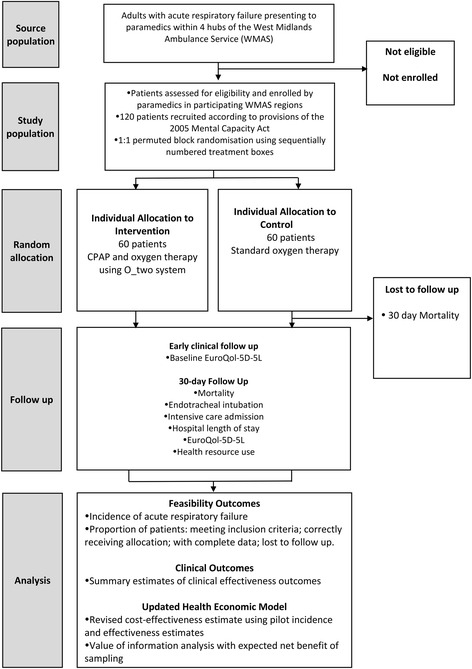
Flowchart of participants through the ACUTE study [CONSORT diagram]

The ACUTE study is a stand-alone, randomised, parallel group, external pilot trial. A concurrent health economic evaluation will also be performed, updating an existing decision analytic model [3]. The study aims to determine the feasibility, acceptability and cost-effectiveness of a definitive trial to evaluate the clinical and cost-effectiveness of prehospital CPAP compared to standard oxygen therapy, for patients attended by ambulance service clinicians with ARF. The study design is summarised in Fig. 1 and the schedule of enrolment, interventions and assessments summarised in Table 1.

**Table 1:** Schedule of enrolment, interventions and assessments

	Study period						
	Enrolment	Allocation	Post-allocation				
Time point	0–5 min	5–10 min	10–60 min	1–5 h	1–29 days	30 days	30–37 days
Enrolment							
Eligibility screen	X						
Informed consent	X				X		
Allocation		X					
Interventions							
CPAP			X				
Standard oxygen therapy			X				
Assessments							
Patient characteristics	X	X	X				
ED physiology and treatments				X			
Hospital treatments					X		
Clinical outcomes						X	
Quality of life and health resource use							X

The primary objectives are to estimate the following feasibility outcomes:

1. The rate of eligible patients per 100,000 population per year
2. The proportion recruited and allocated to treatment appropriately
3. Adherence to allocated treatment
4. Retention and data completeness up to 30 days
5. Expected net benefit of sampling for a range of study sizes to identify the optimal definitive trial design

Secondary objectives are to estimate the following summary clinical outcome measures, across the whole trial population and per treatment group:

1. Proportion surviving to 30 days
2. Proportion undergoing endotracheal intubation by 30 days
3. Proportion admitted to critical care at any point up to 30 days
4. Mean and median length of hospital stay
5. Change in visual analogue scale (VAS) dyspnoea score from presentation to immediately before ED arrival
6. Mean change in quality of life, measured with EQ-5D-5L
7. Key elements of health care resource use up to 30 days

This clinical data will be used to update an existing network meta-analysis and economic model, [3, 9] to determine the cost-effectiveness of prehospital CPAP given current evidence. The summary clinical outcome measures will also inform the design of any future definitive trial.

### Setting and study population

The study will take place in the West Midlands Ambulance Service (WMAS), which serves a mixed urban and rural population of 5.6 million. It employs approximately 4000 staff across five divisions and operates from 15 ‘super-hubs’, each covering five to ten community ambulance stations. Recruitment will take place across four super-hubs in two divisions covering a population of 1.5 million. Included hubs were chosen to provide a representative mixture of urban, semi-urban and rural localities.

The study population will comprise adults transported to hospital by emergency ambulance with ARF. Inclusion and exclusion criteria will be based on ambulance service clinician judgement at the scene of incident. Acute respiratory failure will be defined as respiratory distress with peripheral oxygen saturation below British Thoracic Society (BTS) target levels (88% for patients with COPD, 94% for other conditions), despite supplemental oxygen (titrated low flow oxygen for COPD, or titrated high flow oxygen in other conditions) [13].

Potential participants will be excluded if any of the following criteria are met:

1. Hospital CPAP treatment available within 15 min of eligibility assessment
2. Age < 18 years
3. Known to have terminal illness
4. Known pre-existing lack of capacity (confirmed by relatives, carers or documentary evidence, such as lasting power of attorney)
5. Documented not for resuscitation status
6. Acutely incapacitated patients with known valid advanced directive declining non-invasive ventilation or participation in research
7. The patient has an oxygen alert card
8. Anticipated inability to apply CPAP (e.g. facial deformity)
9. Respiratory failure due to chest trauma
10. Contraindication to CPAP (suspected pneumothorax, respiratory arrest, epistaxis, vomiting, hypotension)
11. Previous enrolment in the ACUTE trial
12. Pregnancy
13. Patients unable to communicate with ambulance service clinicians

### Randomisation, allocation concealment and participant enrolment

Patients presenting to ambulance service clinicians with ARF will be individually randomised to CPAP or standard oxygen therapy in a 1:1 ratio using simple, unrestricted randomisation. The randomisation sequence will be computer-generated by an independent statistician who is not directly involved in the conduct of the trial. The allocation sequence will be held centrally on a password-protected, access-restricted network drive. The trial statistician will not have access to the randomisation sequence until after data lock. Due to the physical differences between the CPAP device and standard oxygen mask, it is not possible to blind patients, ambulance service clinicians or hospital clinicians to the treatment arms. Outcome assessors will also not be blinded. However, both primary and secondary endpoints comprise ‘hard’ objective measurements, minimising the possibility of information bias.

CPAP devices (intervention arm) and high-concentration oxygen therapy masks (control arm) will be packaged in identical equipment boxes measuring approximately 170 mm × 170 mm × 70 mm. The boxes will be tamper-proof and equal in weight and appearance to maintain allocation concealment. Boxes can only be opened after a patient is definitively enrolled in the trial. Once a box is opened when attending a patient, that patient will be included in the study as per allocated treatment. It will not be possible to re-seal the box. Equipment boxes will be assembled, numbered and sealed in accordance with the allocation sequence by researchers not directly involved in the conduct of the trial, under the supervision of the trial manager. Boxes will then be transferred to the central WMAS storage and distribution centre where they will be held in an access-restricted research store. Boxes will subsequently be supplied to participating ambulance hubs as required by the WMAS internal distribution team and held locally in a designated storage area. Ambulance service clinicians will then collect an equipment box at the beginning of each shift. At the end of the shift, boxes will be returned to the ambulance hub equipment store. Boxes will be signed in and out for each shift with ambulance service clinician, ambulance and equipment box details recorded in a distribution log. Research paramedics will monitor the condition, status and location of boxes on a weekly basis to ensure concealment of the allocation sequence until treatment assignment occurs.

Ambulance service clinicians (paramedics, ambulance technicians, paramedic practitioners or critical care paramedics) will identify patients with ARF when attending 999 ambulance calls. Patients meeting trial inclusion criteria will be approached for enrolment in the trial guided by a standardised script. If possible, verbal consent will be obtained for participation prior to enrolment. Patients lacking capacity will be enrolled according to a hierarchical consent process complying with the Mental Capacity Act (MCA) 2005 [14] described below. Immediately after inclusion, ambulance service clinicians will open the trial equipment box and provide treatment according to whether a CPAP device or high-concentration oxygen mask is supplied.

### Consent

A hierarchical consent process will be used complying with the Mental Capacity Act (MCA) 2005 [14]. Verbal consent will be obtained for enrolment if the treating ambulance service clinician determines that the patient has capacity. Ambulance service clinicians will enrol patients without consent if the patient does not have capacity. In either case, a research paramedic will review the participant in hospital as soon as possible after enrolment, provide verbal and written information regarding the study and seek written informed consent as soon as the participant has capacity. If the patient does not regain capacity, we will seek advice from a personal consultee for enrolment in the trial [15]. When a personal consultee is unavailable, a nominated consultee will be approached for a consent waiver [15]. If patients (or their consultees) decline consent, all non-identifiable data up to the point of refusal will be retained. No further data collection will be conducted from this point onwards, except for anonymised 30-day mortality data.

### Trial treatments

Patients in the intervention arm will be treated by CPAP with supplemental oxygen. Patients in the control arm will receive standard oxygen therapy. Treatment in both arms will be targeted to BTS guidelines for peripheral oxygen saturations [13]. Ancillary condition-specific treatments will be administered in both trial arms according to standard practice guidelines [8]. Ambulance service clinicians (paramedics, ambulance technicians, paramedic practitioners or critical care paramedics) will deliver trial treatments.

#### Intervention arm

CPAP is a form of non-invasive ventilation (NIV) where oxygen or air is supplied to the upper airways at increased pressure [16]. The ACUTE trial will use the O_two unit, a lightweight, open, single-use, low-flow CPAP system [17]. The device consists of tubing, which is connected to an oxygen source (either a portable oxygen cylinder or the usual ambulance oxygen flow regulator), and an in-line CPAP unit connecting to a close-fitting face mask. The CPAP unit entrains ambient air to increase local mask pressure, providing resistance for the patient to breathe against. The level of CPAP is varied by altering the incoming oxygen flow rate. Thus, the inspired concentration of oxygen varies according to the desired degree of CPAP, as the flow rate is altered. As an open system, with access to ambient air, the device allows unrestricted inspiratory flows and is unaffected by respiratory rate.

Treatment may be commenced at the site of initial clinical contact or after transfer to an ambulance. An appropriately sized mask will be used, with CPAP started at 5 cm H_2_O and then incrementally increased by 1 cm H_2_O every 2–5 min to a maximum of 15 cm H_2_O according to BTS peripheral oxygen saturation targets [13] measured by standard pulse oximetry. Target peripheral oxygen saturations will be 88–92% for patients with known/suspected COPD and 94–98% for patients with other suspected causes of acute respiratory failure. If necessary, nebuliser treatments can be positioned between the face mask and the O_two CPAP unit. CPAP will be continued until arrival at hospital unless not tolerated (e.g. patient request, claustrophobia, anxiety, significant agitation); patient is unable to maintain own airway; decrease in systolic blood pressure to < 90 mmHg; vomiting; epistaxis; conscious level decreases and patient does not respond to voice; patient improvement; or suspected pneumothorax.

#### Control arm

Oxygen will be delivered at normal atmospheric pressure from a compressed gas tank (or portable oxygen cylinder), via a flow regulator, to the patient using nasal cannula, an air entrainment ‘Venturi’ mask, a simple face mask or a non-rebreathing reservoir face mask. The exact choice of flow rate and oxygen delivery device will be determined by ambulance service clinicians according to the patient’s condition and peripheral oxygen saturation levels. Target oxygen saturations will be 88–92% for patients with known or suspected COPD and 94–98% for other suspected causes of acute respiratory failure.

On arrival at hospital emergency department, staff will be informed of the study and current treatment. Patient care will then be transferred from ambulance service clinicians to hospital clinicians according to normal practice. Care will subsequently continue according to hospital guidelines as implemented by the hospital clinician. In the intervention group, the hospital clinician will determine whether to continue NIV using the O_two unit, switch to an in-hospital system or discontinue NIV altogether. Patients in the control group will be able to receive in-hospital NIV if indicated, according to assessment by the hospital clinician.

The West Midlands Ambulance Service (WMAS), O_two representatives and research paramedics will provide training and support for all ambulance service clinicians based at the participating ambulance hubs. This will involve training in identification of eligible patients, application of the inclusion/exclusion criteria, providing appropriate information and seeking consent, randomisation, delivery of CPAP, monitoring for adverse events (AEs) and data collection. Training will specifically focus on study exclusion criteria, particularly the identification of clinical conditions, e.g. pneumothorax or vomiting, where administration of CPAP could be harmful. Training strategies will include online training modules, small group teaching, demonstration, hands-on familiarisation and scenario-based practice. Only once an ambulance service clinician has received this training and has been assessed to be competent will they be permitted to enrol patients into the trial. Research paramedics will provide ongoing support and training as necessary, including training of new ambulance service clinicians starting at ambulance hubs after recruitment to the study has begun.

### Data collection

A recruitment form (case report form (CRF) A), contained within each equipment box, will be completed every time a patient is enrolled in the trial. This will record study number, basic demographic details, eligibility criteria, suspected prehospital diagnosis,, consent details and limited clinical and treatment data not routinely recorded, including a patient and ambulance service clinician completed VAS dyspnoea scale (1–10) recorded on initial assessment and immediately before ED arrival. Routinely collected baseline characteristics, EMS timings, details of treatments provided and vital signs (including peripheral oxygen saturations) en route to hospital will be extracted from the ambulance service patient report forms/electronic patient records into ACUTE case report form B (CRF B) by research paramedics. At 30 days, research paramedics will also review the hospital records to record details of subsequent progress, treatments provided (including time to receiving hospital NIV, if provided), length of hospital stay, use of critical care, any adverse events (AEs) and status at 30 days. Any related AEs or serious AEs will be recorded on the study case report form (CRF B).

Baseline quality of life assessments will be performed by research paramedics shortly after hospital admission, following confirmation of patient consent for participation in the trial. Patients, or their representatives, will be asked to estimate their current health status, using the EQ-5D-5L. This data will be recorded in the study case report form (CRF B). Quality of life and resource use will also be assessed remotely by questionnaire at 30 days following enrolment. Participants will be asked for their preferred method for data collection, either telephone or postal. Initial non-responders will be contacted again after a further 2 weeks by telephone or post. Key elements of health care resource use to be recorded will include hospital services and GP or community services. Participants will also be asked to report any AEs in the 30-day follow-up questionnaire. For patients’ declining consent for follow-up, anonymised 30-day mortality will be recorded. The assessments and follow-up for the ACUTE trial are summarised in Table 2.

**Table 2:** Summary of data collection and trial documentation

What	Where	Who	How		When	
				Baseline	Hospital admission	30 days
Consent form						
•Verbal consent	Scene of incident	Paramedics	Verbal	X		
•Written informed consent	Hospital	Research paramedic	Paper		X	
Case report form A						
•Patient demographics	Scene of incident/emergency department	Paramedics	Paper	X		
•Patient characteristics			Telephone	X		
•Prehospital treatments •Adverse events				X X		
Case report form B						
•Patient demographics	Hospital	Research paramedic	Paper		X	
•Baseline quality of life					X	
•Inpatient treatments						X
•30-day mortality						X
•Intubation						X
•Critical care admission						X
•Length of stay •Adverse events					X	X
Patient questionnaire						
•Quality of life	Home	Patient	Paper			X
•Resource use		Research paramedic	Telephone			X
•Adverse events						X
Paramedic questionnaire						
•Acceptability of CPAP	Home/work	Paramedics	Electronic	X		
HRA safety report form						
•Unexpected related serious adverse events	CTRU	Chief investigator	Electronic	X	X	X
SAE form						
•Other serious adverse events	CTRU	Chief investigator	Paper	X	X	X

*CTRU* clinical trials and research unit, *SAE* serious adverse event, *CPAP* continuous positive airways pressure

### Data management

All data will be collected and retained in accordance with the UK Data Protection Act 1998 and University of Sheffield clinical trials and research unit (CTRU) standard operating procedures (SOPs). Trial data will be extracted from source documents and CRFs and entered onto a secure data management system. Patient identifiable data (names, date of birth and contact details) will only be collected and entered on the prospect database when written informed consent has been confirmed. Validation reports will be run regularly to check the study data for completeness, accuracy and consistency. Discrepancies will be generated, monitored and managed by research paramedics to resolution.

### Sample size and statistical analyses

Participant recruitment and retention will be presented with a CONSORT flow diagram [18]. The following feasibility outcomes will then be reported descriptively for the whole trial population, together with their 95% confidence interval (calculated using the Wilson score method) [19]:

1. Recruitment rate per 100,000 population per year (target 8, i.e. 120 across the 1.5 million population of the 4 WMAS hubs)
2. Proportion recruited in error and classified as minor or major non-compliances (target 0 and ≤ %)
3. Adherence to the allocation schedule (target ≥ 90%)
4. Adherence to treatment in the CPAP arm (target ≥ 75%)
5. Retention at 30 days (target ≥ 90%)
6. Data completeness (target ≥ 90%)

Summary estimates of effectiveness outcomes will also be reported, for the whole trial population and separately per treatment group, with 95% confidence intervals using an as-randomised analysis:

1. Proportion surviving to 30 days
2. Proportion undergoing endotracheal intubation by 30 days
3. Proportion admitted to critical care at any point up to 30 days
4. Mean and median length of hospital stay
5. Change in VAS dyspnoea score from initial presentation versus immediately before ED arrival
6. Mean EQ-5D-5L
7. Key elements of health care resource use up to 30 days

The ACUTE feasibility study aims to recruit *n* = 120 over 12 months. A minimum sample size of 120 was proposed by Teare et al. for pilot studies with dichotomous outcomes, based on the precision to which binary parameters are estimated for use in the sample size calculation of the full trial [20]. Mortality under standard care is estimated at 12%, and for the full trial, a 5% absolute reduction is postulated (i.e. to 7%) in the intervention arm. With *n* = 120, we will therefore be estimated to within a standard error of 2.7% and used in the sample size calculation for the eventual trial. Given the short follow-up period, loss to follow-up of < 5% at 30 days is envisaged. This sample size will allow estimation of feasibility outcomes with a precision of < 5%.

A previous evidence synthesis study produced estimates of the incidence of eligible cases ranging from 3.5 to 40.8 per 100,000 population per year [3, 9]. The lowest estimates were based upon actual patients treated with CPAP, in services with limited ability to deliver treatment for all eligible patients and are likely to be underestimates. The highest estimates were based upon audit data for in-hospital NIV use among emergency admissions and are likely to be overestimates. Assuming that there are 20 eligible cases per 100,000 population per year and 40% are recruited, 120 patients will be recruited from the study’s source population of 1.5 million, over 1 year.

### Health economics

A previously published meta-analysis and decision analysis model evaluating the cost-effectiveness of prehospital CPAP for ARF will be updated [3, 9, 21]. The decision analysis model simulates the management, outcomes and costs of a hypothetical cohort of patients transported to hospital by emergency ambulance with ARF. Effectiveness is estimated in terms of short-term mortality, using odds ratios from the meta-analysis, and valued as quality-adjusted life years (QALYs) [22]. Costs are estimated from a health service perspective and include all costs related to delivering prehospital CPAP and subsequent treatment of acute respiratory failure. The cost of providing prehospital CPAP is estimated by dividing the total cost of establishing and running the service across an ambulance service by the total number of patients treated.

The ACUTE pilot trial offers an ideal opportunity to estimate the incidence of patients eligible for prehospital CPAP and to update the model with an estimate that is representative and applicable to the NHS. A literature search will also be conducted for new randomised controlled trials comparing prehospital CPAP to standard care; if any are found, the meta-analysis will be updated along with effectiveness data from the ACUTE study. The outputs of the model will be updated estimates of the cost-effectiveness of prehospital CPAP, expressed as the incremental cost per QALY gained by CPAP compared to standard care and the probability of CPAP being cost-effective at £20,000/QALY and £30,000/QALY thresholds for willingness to pay. Expected value of sample information (EVSI) and expected net benefit of sampling (ENBS) for a range of future randomised trial sample sizes will also be calculated. Extensive sensitivity analyses will be performed to explore decision uncertainty including examination of future scenarios where CPAP technology changes in cost or efficacy.

### Trial oversight, ethics and governance

The trial has been reviewed and approved by the NHS Leeds East Research Ethics Committee. The University of Sheffield is providing sponsorship and monitoring oversight for the project, and the trial will be conducted in line with the relevant sponsor SOPs. The Sheffield CTRU is responsible for trial management, oversight of data collection, statistical analysis and the health economics analysis. A Trial Management Group (TMG) comprising the applicants and relevant members of the CTRU team will provide ongoing trial support, have responsibility for interpreting the data and writing and reviewing the final report. An independent Trial Steering Committee (TSC) and Data Monitoring and Ethics Committee (DMEC) have been established to oversee the safety, conduct and progress of the study.

## Discussion

The ACUTE study will determine the feasibility, acceptability and cost-effectiveness of a definitive trial to evaluate the clinical and cost-effectiveness of prehospital CPAP, compared to standard oxygen therapy, for patients attended by ambulance service clinicians with ARF. Recruitment of at least 120 participants to the pilot trial over 12 months will demonstrate that a definitive trial is feasible and cost-effective. It will also allow estimation of adherence, attrition, data completeness and event rates with sufficient precision to ensure validity of the definitive trial protocol. A sample size of 1518 is projected for the full trial (based on 5% absolute effect size, 88% baseline 30-day survival, 90% power, two-sided significance of 5%, 5% attrition at 30 days).

Important design issues during the development of the ACUTE protocol were the choice of CPAP device, method of randomisation/allocation concealment and consent processes. There is a wide array of possible methods for administering prehospital CPAP [16]. A single-use disposable unit was chosen over a CPAP machine on the basis of cheaper cost, simplicity of use and portability. The O_two unit offers several advantages over competing devices. It has low oxygen flow rates, allowing prolonged use without depleting ambulance oxygen stores. It may also be used with portable oxygen cylinders, offering the possibility to commence treatment in a patient’s home. As an open system, with access to ambient air, the device allows unrestricted inspiratory flows and is unaffected by respiratory rate. A potential disadvantage is that the level of CPAP is varied by altering the incoming oxygen flow rate. Thus, the inspired concentration of oxygen cannot be varied independently from the degree of CPAP. The prehospital time interval, and changes in patient physiology (peripheral oxygen saturations, respiratory rate, and dyspnoea score), will be recorded to evaluate any transport delays or prehospital deterioration following introduction of a new EMS treatment. The O_two CPAP device has also been used in Australian and Canadian emergency medical services with an excellent safety record. A range of healthcare practitioners have successfully provided CPAP in these settings (including entry-level technicians, paramedics and critical care paramedics); however, it is possible that effectiveness could vary according to clinician skill and experience.

Cluster randomisation has been used extensively in previous prehospital trials but is limited by a high risk of post-randomisation selection and other biases [23]. Individual randomisation is preferred to maximise internal validity [24] but is challenged by the time-pressured and confused prehospital environment. Central telephone randomisation has not been found to be feasible, and sequentially numbered envelopes have well known limitations. We therefore implemented randomisation using identical trial equipment boxes, packaged according to the randomisation schedule. Packaging was designed to be identical in weight, feel and appearance across trial arms. Research paramedics will closely monitor equipment boxes during the trial to detect any attempts to subvert the randomisation sequence.

Emergency trials will often need to recruit unwell, incapacitated patients. As the most severe ARF patients are most likely to benefit from CPAP, it is not possible to exclude such patients from the trial. In accordance with the Declaration of Helsinki, UK MCA 2005 and Good Clinical Practice guidelines, a hierarchical consent process was designed [14, 25, 26]. Ambulance service clinicians assess capacity as a core skill and will obtain verbal consent for participation if patients can retain, weigh, use and communicate information. In common with other prehospital trials, patients without capacity can be enrolled without consent. At the earliest opportunity after admission to hospital, formal written informed consent will be confirmed with patients or their consultees. These consent procedures were developed in accordance with best practice and after consultation with patient and public representatives, prior to review and approval by an independent research ethics committee.

### Trial status

The current protocol is version 2 (16 February 2017). The current protocol version is available from the study website (www.sheffield.ac.uk/acute). The trial opened to recruitment in August 2017. Recruitment is anticipated to run until 31 July 2018 with trial completion by 31 December 2018. As of December 2017, 25% of the study population was recruited.

## Abbreviations

ACUTE: Ambulance CPAP: Use, Treatment effect and economics
AE: Adverse event
ARF: Acute respiratory failure
BTS: British Thoracic Society
CONSORT: Consolidated Standards of Reporting Trials
COPD: Chronic obstructive pulmonary disease
CPAP: Continuous positive airway pressure
CRF: Case report form
CTRU: Clinical trials and research unit
DMEC: Data monitoring committee
ED: Emergency department
EVPI: Estimated value of perfect information
GCP: Good clinical practice
GP: General practitioner
H_2_O: Water
HRA: Health Research Authority
HTA: Health technology assessment
ICER: Incremental cost-effectiveness ratio
MCA: Mental Capacity Act
NHS: National Health Service
NIV: Non-invasive ventilation
PI: Principal investigator
QALY: Quality-adjusted life year
ScHARR: School of Health and Related Research
SOP: Standard operating procedure
TMG: Trial Management Group
TSC: Trial Steering Committee
UK: United Kingdom
VAS: Visual analogue scale
WMAS: West Midlands Ambulance Service
